# Style Features in Communication of the Crews With Mission Control

**DOI:** 10.3389/fnrgo.2021.768386

**Published:** 2021-11-12

**Authors:** Anna Yusupova, Dmitry Shved, Vadim Gushin, Angelina Chekalina, Natalia Supolkina

**Affiliations:** State Scientific Center of the Russian Federation—Institute of Biomedical Problems, Russian Academy of Sciences, Moscow, Russia

**Keywords:** space flight, crew communication, content analysis, coping strategies, communication styles

## Abstract

In “Content,” an International Space Station (ISS) Russian segment space experiment, features of communication between the cosmonauts and the Mission Control Center (MCC) were studied using content analysis. The method is based on the concept of stress copings by Lazarus and Folkman. Differences found in the communication of cosmonauts led to assumptions about the existence of individual communication styles in routine communication between the cosmonauts and the MCC. The differences found were defined using V. Satir's classical model of communication types. The pre-dominance of three main communication styles (“computing,” “blaming,” and “placating,” as per Satir) was found. Manifestations and features of styles are discussed, considering the effectiveness of the “computing” style for ISS-MCC communication. Cosmonauts with a pre-dominance of this communication style, mostly are stable and with good self-control. An increase of the “blaming” and the “placating” style features in the communication of cosmonauts may require adaptation of the MCC communication and additional psychological support for the cosmonauts.

## Introduction

Crew communication analysis has been common for Russian MCC for more than 40 years, serving to obtain information about the working capacity, mental state, and in-crew interaction of the cosmonauts (Kanas, 2015). A standard daily communication analysis report made by the MCC often includes indications about the individual communicative style of a cosmonaut. To highlight its positive characteristics, a style is often called “constructive, professional” and frequently “corresponding to individual personality traits.” However, behind these expert remarks, there is no common verified classification of communicative styles. We think that style descriptions defining their features would simplify, formalize and, in the future, even help to automate style detection to help formulate precise recommendations to improve and optimize the crew-MCC communication. While individual style detection and analysis for each crew member seems to be excessively difficult to automate, we may suggest that in most cases a typological approach that would focus the attention of MCC experts to problem situations would help provide customized psychological support.

American famous psychotherapist Satir (1972) in her works stated that each person, involved in a closed communicative loop (i.e., family), demonstrated a stable communicative pattern under stressful situations. Satir was the first person who managed to bring together various stable features of communicative styles into the formally described communicative modes (types). Satir indicated that four main communication patterns—placating, blaming, computing, and distracting—occur when “one is reacting to stress and at the same time feels one's self-esteem is diminished.”

“Computing” communication style (Satir, 1972) is expressed in contact by prudence, precision, and desire to analyze the situation, to collect the maximum amount of data not to make a mistake. We suggested that a cosmonaut with a dominant computing style would be constantly searching for information and giving feedback to the MCC, planning his activity on its basis, and using self-control–coping strategies, without evident emotionality in negotiations with the MCC. In discussions, this style would manifest itself in clear rationales for decisions—rational rather than emotional expressions of agreement and disagreement. This style has the potential necessary for successful problem resolution based on effective information exchange and accurate and balanced decisions. The disadvantages of the style include impersonality, semantic narrowness, difficulties in transferring emotions (that is necessary under stress), and accepting psychological support.

“Blaming” communication style (Satir, 1972) is based on the intention to create a psychological defense against possible guilt, to counterattack, thereby demonstrating readiness to cope with the situation, to take control over it by means of disagreements and accusations. From the point of view of the subject, the problem is not so much caused by the objective factors, but by the partners involved. Thus, “blaming” communication patterns would lead, at best, to psychological defense reactions such as reciprocal demands, counter-proposals, distrust, even to claims, confrontation, and refusals to collaborate. The positive side of this style is the intention of the “blamer” to analyze the situation quickly, find an external cause, and offer an option for problem-solving. The negative side is slipping into the search for another guilty instead of discussing the problem. In accusations, excessive generalizations and negative emotions may appear. Thus, in “blaming” communication, the social regulation and the affective components grow, and the pure data exchange in the contact decreases.

Describing the “placating” pattern, Satir (1972) emphasized the desire of the subject to be guided in the behavior by the “important others” opinion about himself and to avoid confrontation at any cost (e.g., by taking responsibility and even expressing guilt for what happened). In a problem situation, the “placater” often claims that he is experiencing difficulties without external support. At the same time, he is ready to take responsibility for mistakes, to agree when criticized. This communication style might be expressed in coping strategies such as seeking support, making requests, excessively informing others in order to obtain recommendations, as well as acceptance of responsibility, and self-justification. The advantages of this style may include such intentions as providing partners with a large amount of information to allow them to control the process from the outside, strict execution of orders and recommendations, and the desire to avoid conflict. The disadvantages of “placating” may include lack of initiative, excessive dependence on partners, insecurity, and even anxiety. Communication of the “placater,” as in the case of “blaming,” might be emotional and focused on social regulation from the outside. At the same time, the data exchange component is expressed in their communication very actively.

Finally, the “distracting” pattern is associated (Satir, 1972) with the “nobody cares about me” attitude. A person using this style tends to devalue what is happening—in order to relieve himself from the responsibility of solving the problem. Its manifestations are associated with the use of coping strategies of the “distracting” group in communication: minimization of contacts, a small number of answers to the essence of the question, emotional distancing, mistrust, and responsibility avoidance. We supposed that this destructive style would rarely manifest itself in the communication of astronauts due to its inconsistency with flight tasks and strict selection procedures.

We consider that the classification of communicative styles by Satir perfectly matches our research needs. The first reason for this choice is that Satir's model is addressing communicative behavior under stress, that corresponds to the prime target of the psychologists of Institute of Biomedical Problems (IMBP): starting from the 1970s, they were analyzing communication in the close loop crew-MCC (also a sort of the “family”) to obtain information about the psychological health of the cosmonauts under space stress (Myasnikov and Simonov, 1982; Myasnikov and Stepanova, 2000). Feeling lack of trust and unwillingness of the specialists of the MCC to hear their position (Beregovoi et al., 1993) was considered as the main communication problem by certain cosmonauts who gave interviews after a flight (in the frame of “Content” space experiment). The second reason to choose Satir's model was that it focused on the sources of distortion in data exchange during contacts in close systems that correspond to the communication concept of Lomov (1981), one of the famous Russian psychologists who worked on the communication theory. Both Satir and Lomov discussed the ways of problem-solving facilitation during contacts and detection of moments when pure data exchange is influenced by stable personal patterns, such as discussion of current subordination (social regulation function of communication, as per Lomov) or expression of experienced emotions (affective function of communication). The last reason is that Satir's model is attractive for its simplicity: for non-specialists, it is easy to understand, remember, and recognize by the MCC personnel. It is also very practical, as a psychotherapist Satir built her communication model for practical use, to detect stress-connected communication patterns that need correction. The early identification of these patterns is what we intend to use in spaceflight communication analysis for early stress detection and consequent psychological support, targeted at the rise of the self-esteem and stress reduction of the astronauts.

That is why we applied this model to build a methodology for “Content” space experiment data analysis (Gushin et al., 2016), where the main assumption was that stressful situations in flight lead to the manifestation of coping strategies [as per Lazarus and Folkman (1984)], that is focused on problem-solving and stress reduction. Based on this assumption, at the initial stage of the study, in the communication of each cosmonaut, we identified a dominant attitude to the stressful problem and coping strategies associated with each style, as per Satir. In addition, we assumed that in the talks of the cosmonaut with the MCC during the prolonged flight, depending on his mental state and the situation on board, several styles could be present simultaneously, with some of them dominating in each period.

## Methods

Our content analysis method is based on counting the number of statements (verbal actions done to express an opinion, which may consist of several sentences) in the talks of cosmonauts with the MCC related to the categories under study (coping strategies and operational categories). In 2014–2015, we made a pilot pre-study to check intercoder reliability: the consistency of four independent coder opinions was assessed using the Spearman's rank correlation method [the procedure is explained in detail in Gushin et al. (2020)]. The opinions of each expert on 19 assessment criteria (Table 1) were compared to the group opinion. We used the graphical median method to calculate the group raw score for each category. The final agreement coefficients for each expert and group turned out to be reasonably high (rs = 0.76–0.89) to consider the technique reproducible and valid.

**Table 1:** Content analysis categories used in study.

Problem-oriented coping strategies	Effective	- Planning, scheduling -Initiative
	Ineffective	- Confrontation - Critique - Responsibility avoidance - Rational refusal to perform
	Ambivalent	- Subordination - Taking responsibility - Seek for support
Emotion-oriented coping strategies	Effective	- Self-control - Positive reassessment - Emotional support -Humor
	Ineffective	- Mistrust - Ignoring the problem, avoidance
Operational categories		- Informing - Time management - Requests/demands -Sleep

In the space experiment, we were searching for the features of a dominant communication style in each cosmonaut that might have been considered as an individual speech behavior norm. In the crew-MCC communication, we counted the categories that manifest (according to coder experts) stress-coping communication strategies. The concept of the manifestation of stress through coping strategies was proposed by Lazarus and Folkman (1984), who showed that coping strategies are emotional, motivational, cognitive, and behavioral constructs that manifest themselves in all types of activities, including speech. Coping strategies are used by people to adapt to situations that require psychological resilience to reduce the level of stress experienced. One of the generally accepted ways of categorizing coping strategies is to divide them into two groups (Lazarus and Folkman, 1984): problem-oriented, aimed at solving the problem of distancing the subject from the stressor (problem), and emotionally oriented, aimed at making the problem less psychologically painful, more bearable (Table 1). We should note that the same coping strategy in different situations can simultaneously be aimed at solving problems and reducing emotional stress (Lazarus and Folkman, 1984).

Since one of the tasks of the “Content” space experiment is to study the behavior of the cosmonaut under stress, the coding task was to count statements attributed to preliminarily defined operational categories and stress-coping strategies manifestation in the conversations of cosmonauts with the MCC. Then we grouped these coping strategies according to Satir's communication model to determine the dominant style of each subject. To interpret the content analysis data, we also used the weekly psycho-neurological reports of the MCC and the data of post-flight interviews. The content analysis data were compared with the results of the weekly neuropsychiatric conclusions of the crew psychological support group (Yusupova et al., 2019).

The subjects were male cosmonauts of ISS 43/44–54/55 flights, who took part in the “Content” space experiment, *N* = 15, age-range 40–57 years. Among these cosmonauts, seven subjects made 1 or 2 flights, and eight subjects made 3–6 flights.

A corpus of ~1,64,658 statements containing categories of interest was selected from official Roscosmos transcriptions made daily for open (non-confidential) communication channels. All subjects signed informed consent for participation in the “Content” experiment. The data were normalized as the rate of statements per week for analysis and processed using SPSS-2018 software, methods used were principal component factor analysis (Varimax rotation method with Kaiser normalization), Kruskal–Wallis one-way ANOVA, and Mann–Whitney U test. The choice of the non-parametric criterion was due to the fact that in normality check for all data variables (categories of content analysis), a pronounced skewness (to the right) and kurtosis were detected.

## Results

Expert coders (psychologists and psychotherapists) made separate independent opinions to attribute the communication features of individual cosmonauts to Satir's styles in accordance with the three groups of criteria that are defined (described above in the Introduction section). These opinions were compared and discussed to make a common decision about the division of subjects (*N* = 15) into groups. Coders agreed to form three groups: four subjects with attributed the “computing” (~29,499 statements), four with “blaming” (~55,287 statements), and three with the “placating” style (~56,231 statements). Four subjects were unattributed to the three groups.

**Figure 1:**
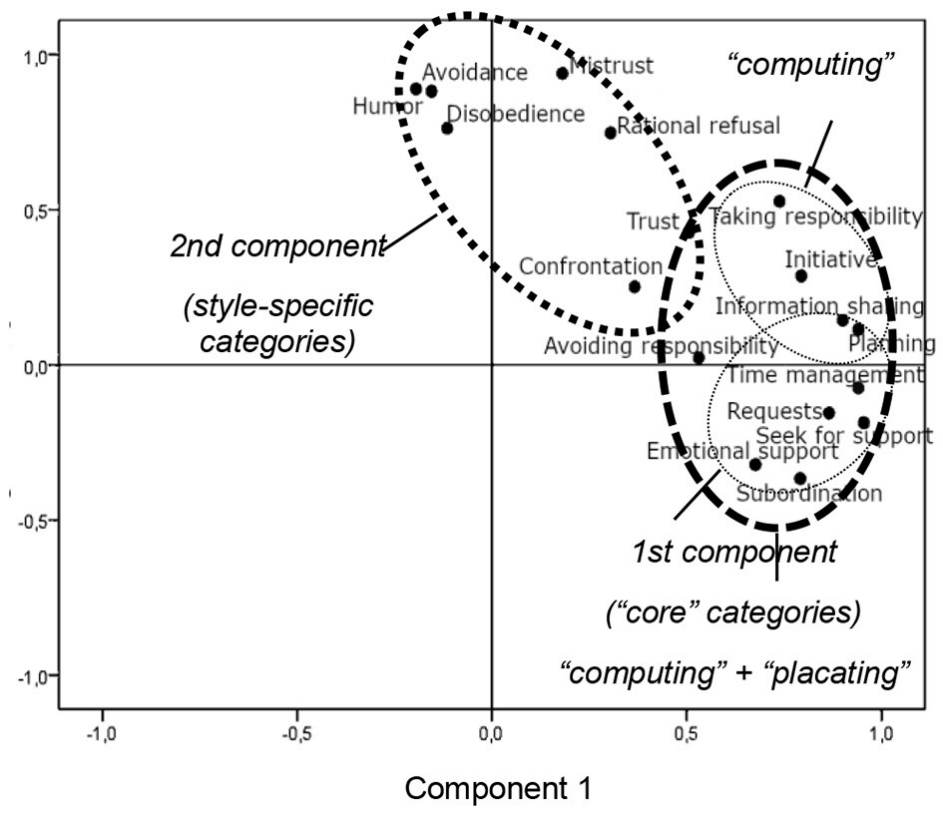
Identification of significant categories using the method of principal components.

Factor analysis carried out at the first stage of the experiment made it possible to define groups of content analysis categories (Figure 1). The first component included the following categories: Taking responsibility, Initiative, Information sharing, Planning, Avoiding responsibility, Time management, Requests, Seek for support, Emotional support, and Subordination. The second component included Humor, Avoidance, Mistrust, Disobedience, Trust, and Confrontation. This analysis defined the groups of content analysis categories that seem most significant and adequate to the research objectives. The first component included categories most frequently used in the speech of cosmonauts and the second component included categories that were unusual for the crew-MCC communication pattern.

We used the style splitting to search for differences in the use of communication features in three style groups using Kruskal–Wallis one-way ANOVA (**Table 3**). “Computing” style cosmonauts express less Support categories compared to the “placating” and the “blaming” style cosmonauts (*H* = 5.326; df = 2; *p* = 0.07). Support category is most common among the “placaters.”

“Computing” style cosmonauts have the lowest values in the Demands/Requests category, compared to the “placaters” and the “blamers” groups (*H* = 6.962; df = 2; *p* = 0.031). This category is most common in the “placaters” group.

The Subordination category also has the lowest average values for “computing” cosmonauts, compared with the “placaters” and the “blamers” groups (*H* = 5.598; df = 2; *p* = 0.061). The highest average values for the Subordination category were found in the “placaters” group.

Humor category averages were highest in the “blamers” group, compared with two other groups (*H* = 5.386; df = 2; *p* = 0.068). At the same time, the average values for the “placaters” and the “computers” have a barely noticeable difference.

The “computing” style group had the lowest averages in the Responsibility avoidance category, compared to the “placaters” and the “blamers” groups (*H* = 5.326; df = 2; *p* = 0.007). The “placaters” have the highest average values in this category.

The highest mean values for the Self-control category were found in the “blamers” group compared to the other two groups (*H* = 5.667; df = 2; *p* = 0.059). The lowest average values for this category were found in the “computing” style group.

The highest mean values for the Trust category were found in the “blamers” group, while average values for this category were equally low in the “blamers” and the “computers” groups (*H* = 7.053; df = 2; *p* = 0.029). Also, “blamers” had the highest mean values in the Rational refusal to perform category, compared to the “placater” and the “computer” groups (*H* = 5.386; df = 2; *p* = 0.068). The cosmonauts assigned to the “computing” style had the lowest average values in this category.

## Discussion

Factor analysis distinguished two large groups of categories of statements that were present in the communication of crew members (as shown in Figure 1). The main core of the crew-MCC communication includes categories that are the most frequent in cosmonauts' speech: Informing, Planning, Time management, Subordination, Initiative, Requests, and so on. Communication between the crew and the MCC is mainly based on informing the MCC about the state of work and initiatives of the crew, discussing the application of the recommendations, and requests for information support received. When problematic situations arise, the topics of statements related to the responsibility distribution are also relevant.

The second component aggregates categories of statements that deviate from standard crew-MCC communication pattern—most of them correspond to the “blaming” communication style. These are Confrontation, Rational refusal to perform, and Mistrust. At the same time, subjects expressing the “blaming” style, were not using only second component statements, but also categories from the “communicative core” as well, such as Initiative and Taking responsibility.

The categories attributed to “computing” and “placating” styles are close in the factor space, mixing with what we can call a “communicative core” of the standard crew-MCC communication. However, “computing,” in accordance with our preliminary hypothesis, is also characterized by closely spaced categories of statements related to Informing, Planning, and Initiative, while for “placating,” as we expected, we can identify statements related to Seek for support, Subordination, and Requests.

We should emphasize that the “placating” style of communication in our sample was mostly typical for new cosmonauts (maybe we should call it a “beginner style”). Therefore, it may not be an inherent, personal style of communication, but based on the lack of experience.

**Figure 2:**
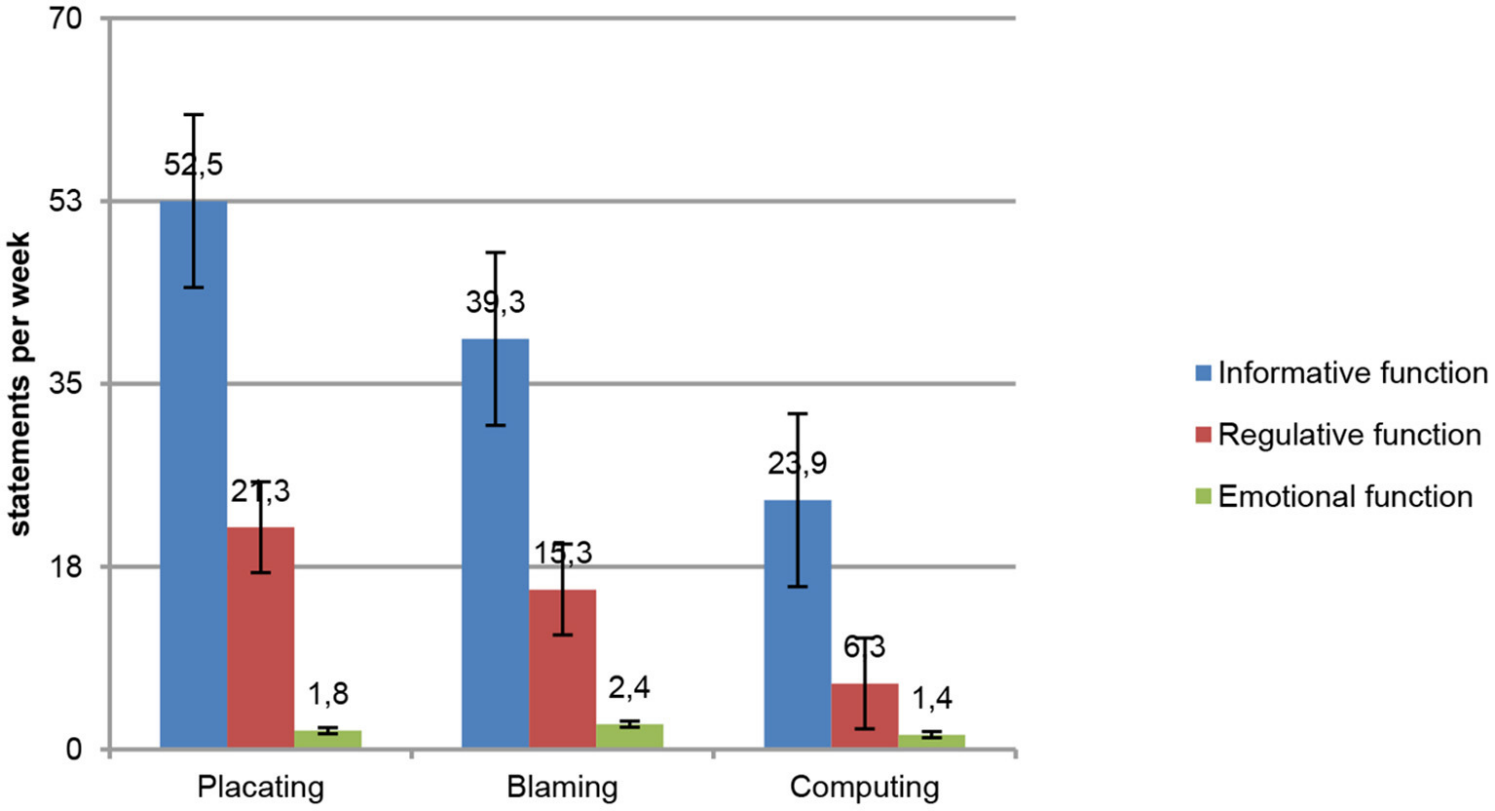
Distribution of content analysis categories average weekly use in style groups by Lomov (1981) three communication functions.

Coping strategy categories were conditionally divided according to Lomov (1981) communication functions theory (data sharing, social regulation, and affect) for further calculation of how much these three categories are presented in cosmonauts' communication with MCC (as shown in Table 2 and Figure 2). In our previous research (Gushin et al., 2020), we analyzed the use of coping strategies in different flight stages, but the current study is focused on communication styles.

**Table 2:** The main categories of content analysis and communication functions, as per Lomov (1981).

**Communication functions**	**Content analysis categories**
Information	Informing/information sharing
	Requests/demands
	Time
	Planning/scheduling
	Initiative
	Rational refusal to perform
Social regulation	Subordination
	Critique
	Denial, ignoring the problem
	Seek for support
	Responsibility avoidance
	Confrontation
	Mistrust
Affect	Humor
	Self-control

The “computing” style manifested itself in regular reports to the Earth about what is happening onboard (Informing), combined with clarifying questions to specialists before making decisions. The solution of problems was carried out through their awareness and unambiguous “understanding” (this word is the most frequent verbal manifestation of this style, its semantic marker). Informing statements prevailed in speech, subjects showed readiness to follow the plan (Subordination) while being Initiative, questioning the rationality of Time use, and constantly Planning. In problem situations, agreement or disagreement with the position of the MCC was rationalized; explanations were made in an emotionless manner. Three out of eight cosmonauts with “computing” style had minimum manifestations of Confrontation coping.

Distribution of content analysis categories by Lomov's three communication functions (Lomov, 1981) shows the expected dominance of the information function in subjects with a more pronounced “computing” style (Figure 2). In the “computing” style, there are a lower number of statements for social regulation categories (Subordination, Responsibility avoidance, Confrontation, etc.): this corresponds to Satir (1972) idea about the lesser importance of socially significant interactions for this communication style. Although the absolute value of informative messages in the “computing” group was lower than that in others (Figure 2), the share of the “informing” category in communication was higher than for the “blamers” and the “placaters.” The total volume of communication (number of statements) in “computers” was lower than in others, due to the low levels of statements about the distribution of roles and the manifestation of emotions (two other functions of communication).

These results confirmed the initial supposition about the prevalence of the “computing” style (based on the maximization of dry data exchange) in the communication of the cosmonauts with the MCC. Considering communication with the MCC in flight as an element of professional activity may lead to the development of professional communication skills, including certain coping styles: precise Informing about what is happening at the station and accurate Scheduling (Planning) based on obtaining clarifying information. Regarding the dominance of the time-saving “computing” style in the studied group, we can assume this style to be the most effective to resolve the existing inflight problems relying on the support from the MCC.

The second most frequent communication style detected was “blaming.” Distinctive features of this style are the intention to take control over problems by finding someone else responsible and proposing their solutions. Four subjects were included in this group. In routine communication, cosmonauts expressing “blaming” style, after a quick analysis of the problem (made with a certain irony), made counter-proposals and expressed their Initiatives for correcting schedules (Time category). In this group, although the overall communication volume and Informing statements were higher than in the “computing” group, Confrontation, Refusals, and Mistrust were also present. In the worst cases, “blaming” communication led to emotional Confrontation and expression of Mistrust in the competence of the interlocutor. Verbal markers of “blaming” were confrontational questions, like: “Who did that …?,” “Are you kidding me?,” “How long is it going to last?” Along with Confrontation, we found irony and sarcasm typical for blaming. This reaction is similar to what was described by several authors [e.g., Kanas (2015)] as emotional transfer—a form of psychological defense allowing a cosmonaut to stabilize his psychological state through draining of negative emotions accumulated during a long-term flight (Suedfeld et al., 2015). “Complaints” in crew talks were often objectively justified by chronodeficiency in MCC plans, experts' misunderstanding of the project's place in the overall structure of the schedule, inaccurate description of procedures and conditions made by specialists, equipment availability aboard, and so on (Myasnikov and Stepanova, 2000). But according to MCC psychologists, sometimes the manifestations of this style were related not to the real difficulties of the situation itself, but the level of stress experienced by the crew member—and that could be a point of attention for the psychological support team.

The “placating” style (Satir, 1972), noted in three subjects, was more common among young cosmonauts who made their first or second flight. The members of this group communicated with the MCC more than their experienced colleagues, informed specialists about what was happening onboard more often, seeking to obtain approval of what they were doing (Subordination and Seek for support categories). Thus, the overall communication volume of the “placaters” was the biggest (Figure 2). Subjects with the “placating” style also experienced a lack of Time for flight tasks completion more frequently (Table 3). The verbal markers of “placating” that we detected were the words “help” and mentions of “lack of time.”

**Table 3:** Kruskal–Wallis one-way ANOVA, grouping variable: styles.

**Category**	**Placating**	**Blaming**	**Computing**	**H**	**df**	**Asymptotic value**	**Eta squared**
Support	10.33 ± 4.99	9.63 ± 9.73	2.4 ± 2.29	5,326	2	0.07	0.38
Requests/demands	11.71 ± 5.12	6.81 ± 3.66	3.37 ± 1.15	6,962	2	0.031	0.624
Subordination	13.65 ± 5.94	5.86 ± 3.00	4.03 ± 0.79	5,598	2	0.061	0.667
Humor	0.64 ± 0.29	2.48 ± 1.94	0.66 ± 0.86	5,386	2	0.068	0.438
Responsibility avoidance	2.83 ± 1.42	1.09 ± 0.64	0.64 ± 0.37	5,326	2	0.07	0.67
Self-control	1.15 ± 0.57	1.84 ± 0.43	0.76 ± 0.24	5,667	2	0.059	0.64
Trust	0.25 ± 0.009	0.83 ± 0.28	0.23 ± 0.22	7,053	2	0.029	0.73
Rational refusal to perform	1.24 ±+0.63	1.66 ±+0.9	0.43 ± 0.29	5,386	2	0.068	0.502

So, the “placating” style as we found in our sample manifested itself in unquestioning Subordination (based on a lack of confidence and experience) and excessive Informing of the MCC and the specialists. In a frequent effort to obtain confirmation of the correctness of their actions, they provided the MCC with a greater amount of information about what was happening at the station. In return, they got approval and Support. In problem situations, crew members demonstrating a “placating” style tried to avoid Confrontation and Responsibility for mistakes, justifying the complexity of tasks and the Time deficiency.

## Conclusions

The differences revealed in the communication of the cosmonauts with the MCC made it possible to make assumptions about their communicative styles based on Satir (1972) model.

1. Formalization of the quantitative criteria of the cosmonaut's communication style makes it possible to clarify the boundaries of his norms. Their understanding may help the MCC in personalization of contacts with crew members in flight and elaboration of proper communication strategy. This can increase the efficiency of data exchange, help to avoid confrontation, and provide better psychological support.
2. The more the “informing” function dominates in contact, the more effective the communication session is for resolving operational problems. Therefore, the “computing” style, that corresponds to the norms of effective business relations, based on clear regular data exchange with MCC and subordination to specialists' recommendations, dominates in the space crews' communication with earth.
3. Cosmonauts' communication with mcc allows not only sharing the data but also feelings and attitudes, so their communication styles reflect individual strategies of stress coping in emerging problem situations. Therefore, an increase in the manifestations of the “blaming” and the “placating” styles may be an early sign of distress that requires the MCC to modify the communication style in its turn, as well as execute additional psychological support.

## Data Availability Statement

The raw data supporting the conclusions of this article will be made available by the authors, without undue reservation.

## Ethics Statement

The studies involving human participants were reviewed and approved by Institute for Biomedical Problems TSNIIMASH Roscosmos Corp. The patients/participants provided their written informed consent to participate in this study.

## Author Contributions

All authors listed have made a substantial, direct and intellectual contribution to the work, and approved it for publication.

## Funding

This study was supported by the Russian Academy of Sciences (63.2).

## Conflict of Interest

The authors declare that the research was conducted in the absence of any commercial or financial relationships that could be construed as a potential conflict of interest.

## Publisher's Note

All claims expressed in this article are solely those of the authors and do not necessarily represent those of their affiliated organizations, or those of the publisher, the editors and the reviewers. Any product that may be evaluated in this article, or claim that may be made by its manufacturer, is not guaranteed or endorsed by the publisher.

## References

[B1] Beregovoi G. T. Bogdashevskij R. B. Grigorenko V. N. Pochkaev I. N. (1993). Space Academy (In Russian). Masinostroejenie.

[B2] Gushin V. Shved D. Vinokhodova A. Bubeev Y. S?hastlivtceva D. Yusupova A.. (2020). “Selected Russian contributions to spaceflight, Part 2: Russian space experiment “CONTENT”,” in Psychology and Human Performance in Space Programs: Extreme Application, eds Landon L. B. Slack K. J. Salas E. (Boca Raton, FL: CRC Press), 284-289.

[B3] Gushin V. Yusupova A. Shved D. Shueva L. Vinokhodova A. Bubeev Y. (2016). The evolution of methodological approaches to the psychological analysis of the crew communications with Mission Control Center. REACH. 1, 74–83. 10.1016/j.reach.2016.05.001

[B4] Kanas N. (2015). Humans in Space. New York, NY: Springer. 10.1007/978-3-319-18869-0

[B5] Lazarus R. Folkman S. (1984). Stress, Appraisal and Coping. New York, NY: Springer.

[B6] Lomov B. F. (1981). Problem of Communication in Psychology (In Russian). Moscow: Nauka:

[B7] Myasnikov V. I. Simonov P. V. (1982). Distant Observation and Expert Assessment (In Russian). Moscow: Nauka.

[B8] Myasnikov V. I. Stepanova S. I. (2000). The problem of mental asthenization in prolonged space flight (In Russian). Moscow: Slovo.

[B9] Satir V. (1972). Peoplemaking. Palo Alto, CA: Science and Behavior Books.

[B10] Suedfeld P. Brcic E. Johnson P. J. Gushin V. (2015). Coping strategies during and after spaceflight: data from retired cosmonauts. Acta Astronaut. 110, 43–49. 10.1016/j.actaastro.2014.12.011

[B11] Yusupova A. K. Shved D. M. Gushin V. I. (2019). Preliminary results of “content” space experiment. Hum. Physiol. 45, 710–717. 10.1134/S0362119719070181

